# Incidental whole-body MRI evidence of COVID-19 in an asymptomatic patient in a high prevalence region

**DOI:** 10.1186/s43055-020-00288-x

**Published:** 2020-09-09

**Authors:** Valentina Angelini, Alberta Villanacci, Angelo Belotti, Francesca Castagnoli, Barbara Frittoli, Antonio Corvino, Arturo Brunetti, Luigi Grazioli

**Affiliations:** 1 Dipartimento di Oncoematologia, Diagnostica per Immagini e Morfologica e di Medicina Legale, Università degli Studi di Napoli “Federico II”, Via Pansini, 5, 80133 Naples, Italy; 2 ASST “Spedali Civili di Brescia”, Dipartimento di Radiologia1, Piazzale Spedali Civili 1, Brescia, Italy; 3 Dipartimento di Radiologia, Università degli Studi di Brescia, 1 Brescia, Italy; 4 Motor Science and Wellness Department, University of Naples “Parthenope”, Via F. Acton, 38, I-80133 Naples, Italy

**Keywords:** COVID-19, Whole-body MRI, Incidental findings, Chest CT

## Abstract

**Abstract:**

**Background:**

The purpose of this case report is to emphasize the importance of curing any clinical radiological elements in this historical period, especially in the area of endemic to coronavirus disease 19 (COVID-19) such as Lombardy and to stress the importance of the management of the asymptomatic patient, their crucial role in the spread of contagion.

**Case presentation:**

We reported the case of incidental diagnosis of interstitial pneumonia by first finding on whole-body MR (WB-MR) in the patient affected by multiple myeloma (MM), with a negative respiratory symptoms at the time and with previous (1 month before) negative chest X-ray. The patient was promptly subjected to chest CT, which confirmed the suspicion of interstitial COVID-19 pneumonia and, in hospitalization, performed nasopharyngeal swabs for real-time polymerase chain reaction (RNA-PCR), with a doubtful outcome. Once the bacterial nature of the alterations was serologically and radiologically excluded, the patient was definitively diagnosed with COVID-19 and appropriately treated in hospitalization.

**Conclusion:**

The clinical choices must, therefore, to make use of all the diagnostic tools available and full knowledge of the limitation of each of them.

## Background

SARS-CoV-2 is a novel coronavirus isolated in the respiratory tract of patients with infection of unknown causes on December 31, 2019, in Wuhan, Hubei, China [1–3], and consequently, it has spread globally, becoming a pandemic. In Europe, this infection had a high prevalence and a high infection rate in Northern Italy, even if the causes are currently undefined. It belongs to the family of *Coronaviridae* that includes viruses that cause diseases ranging from the common cold to severe respiratory syndromes (SARS) and the Middle East respiratory syndrome (MERS).

The most common COVID-19 symptoms are fever (37.5-38 °C), dry cough, dyspnea, myalgia, syncope, and fatigue [4–6]. Some studies reported gastrointestinal involvement (intestinal ischemia, cholecystitis), acute cardiac, kidney injury [6, 7], and neurological manifestations (dizziness, headache, hypogeusia, and hyposmia) [8]. The most severe patients had a sudden development of ARDS (acute respiratory distress syndrome white lung at chest RX) ischemic or hemorrhagic stroke, loss of consciousness [8].

Many subjects, however, may be initially or throughout the infection completely asymptomatic and they are above all the contributors to propagate infection in the population. So far, the possibility of contracting COVID-19 infection is considered most likely in patients with fever and/or respiratory tract symptoms who have had close contact with a suspected or confirmed affected patient RT-PCR and next-generation sequencing have been used for the definitive diagnosis of this new coronavirus [4], according to the indication of WHO.

The RT-PCR test for COVID-19 is well known for its high specificity; however, it has a low sensitivity (60-70%) [9, 10] as well as a great variability related to the moment of performing the swabs (prodromal phase or full-blown phase) and area analyzed (nasopharyngeal swab is more reliable than oropharyngeal). Besides, the constant growth in number of swabs to be analyzed had as a consequence the delay of results.

Therefore, a further technique with higher accuracy needs to be implemented to improve the management of patients admitted to hospitals during the pandemic. Radiologists can help in this task by identifying and characterizing the pulmonary involvement of COVID-19 [11]. CT typically shows bilateral ground-glass opacities (GGOs), with a predominantly peripheral, subpleural location [12–14]; intralobular reticulations can be seen superimposed on the ground-glass opacities, resulting in a crazy-paving pattern which is usually associated with a more severe stage of the disease. In the high prevalence region, the radiologists could help to individuate incidental thoracic alterations, too, especially in asymptomatic patients.

This article reports the case of accidental diagnosis of the first finding of COVID-19 by WB-MR of a patient with MM. The patient had no respiratory symptoms at the time of the examination and 1 month earlier had a negative chest X-ray.

## Case presentation

The patient is a 60-year-old male affected by IgG lambda MM in complete remission after ASCT performed in February 2018, followed by continuous maintenance therapy with oral immunomodulating agent lenalidomide. Since the beginning of pandemic, the daily activity in our radiology department has been deliberately reduced in order to avoid infections and contribute to the lockdown started in Italy on 7 March 2020. Before entering the hospital facilities, each patient must necessarily perform diagnostic tests, must undergo the measurement of temperature, and must wear a mask and gloves, as well as medical, technical, and nursing staff. After every MR exam, the machine is sanitized.

**Fig. 1:**
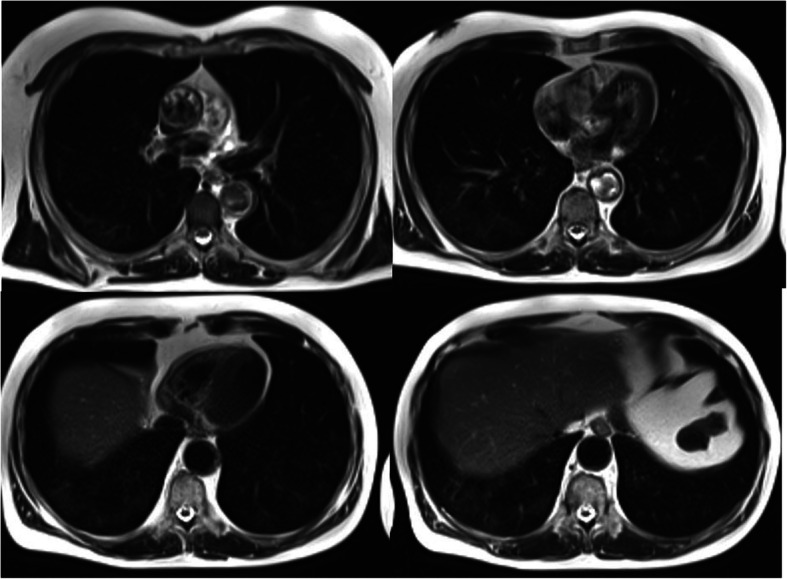
RM- Whole Body, T2-w sequences on the thorax. There are peripheral thickenings of both lungs

**Fig. 2:**
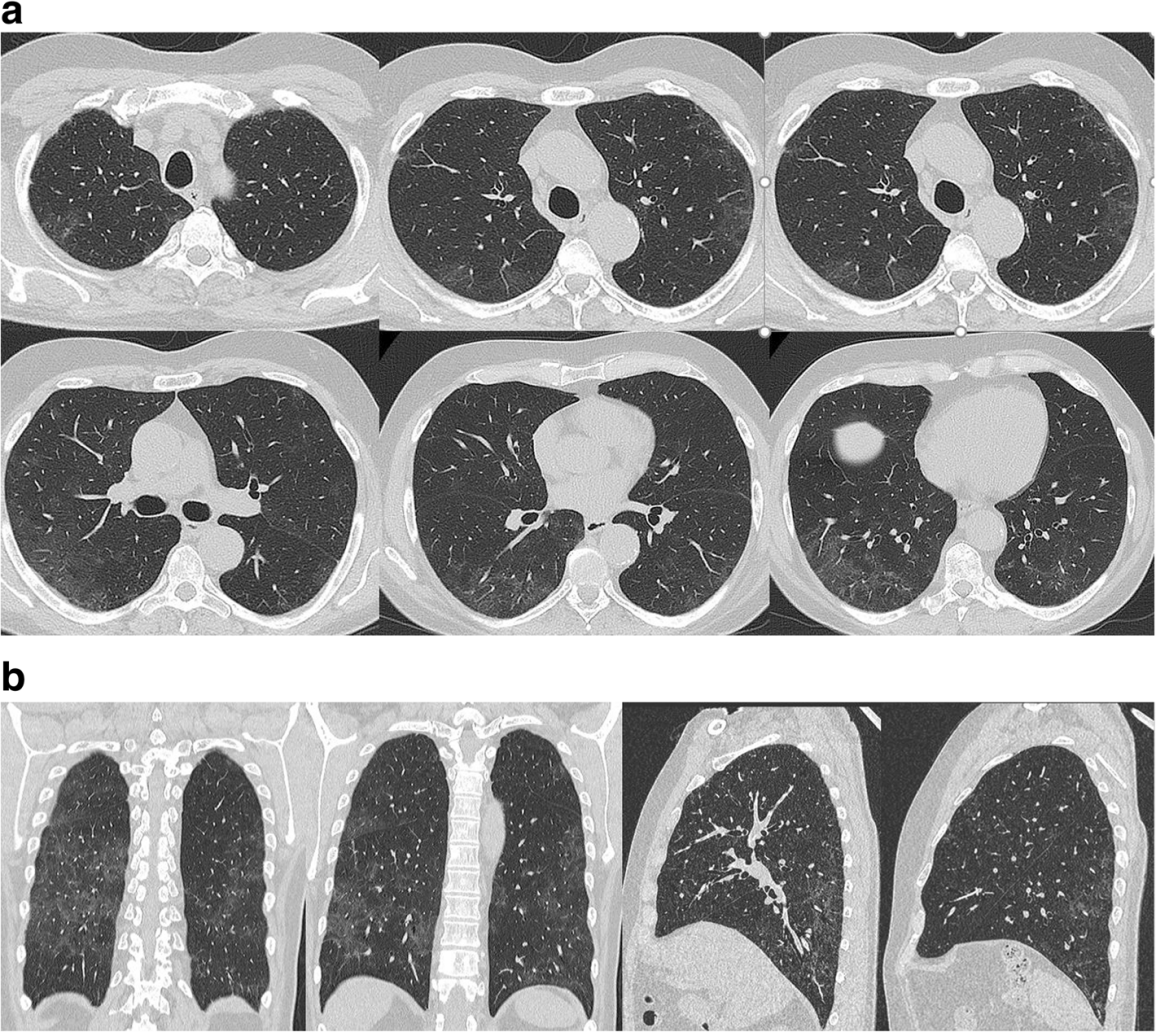
Chest CT. In (**a**) on axial floor. In (**b**) coronal and sagittal floor. There are multiple bilateral peripheral ground-glass areas, more evident at basal and right, but present at the top of lungs too associated with the crazy-paving pattern, with the prevalence of interlobular e intralobular thickening compared to the ground-glass pattern. There are no evident pulmonary consolidations and/or mediastinal lymphadenopathies

On April 10, the patient performed WB-MR planned for the radiological follow-up of his MM while he was completely asymptomatic. On the T2w sequences and the T1w inspiratory on the thorax, peripheral thickenings of both lungs were highlighted (Fig. 1) so a chest CT was required to integrate. Latter chest CT showed multiple bilateral peripheral GGOs, more evident at basal and right, but present at the top of lungs too associated to crazy paving pattern, with prevalence of interlobular e intralobular thickening compared to GGO pattern. There were no evident pulmonary consolidations and/or mediastinal lymphadenopathies (Fig. 2a, b). These alterations were all of new onset (the examination was compared with a previous skeleton CT, thus excluding chronic interstitial disease) and they were suggestive for COVID-19.

**Fig. 3:**
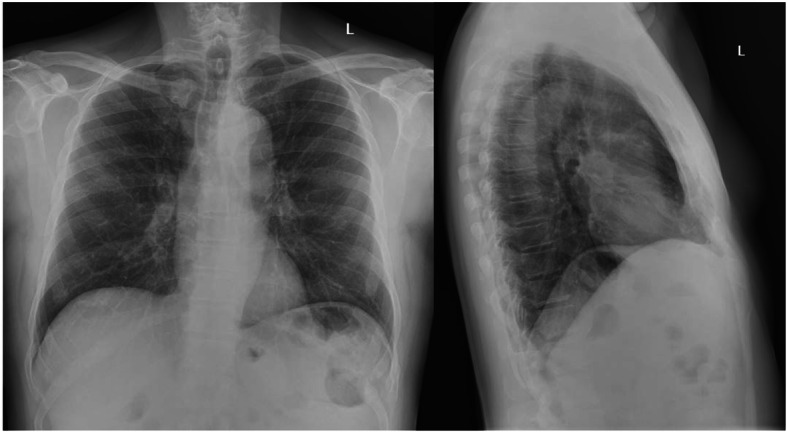
Chest-RX, PA, and LL projections (March 13). No evidence of pleuro-parenchymal alterations in both lung areas

The patient was clinically re-examined and he reported that on March 13, he went spontaneously to the emergency department of the same hospital structure due to a slight, but constant weakness, so he had already undergone a chest X-ray that was negative (Fig. 3).

On March 16, amoxicillin therapy for the occurrence of fever and cough was started and lenalidomide therapy was temporarily interrupted. Fever resolved 5 days later but the mild cough persisted for the following week until March 26. However, due to the concomitant COVID-19 local outbreak, he observed home isolation for 2 weeks.

In light of these new anamnestic data and of the radiological suspicion of COVID-19, despite the asymptomatic state of the patient, nasal pharyngeal swabs were performed for RT-PCR and he was hospitalized.

**Fig. 4:**
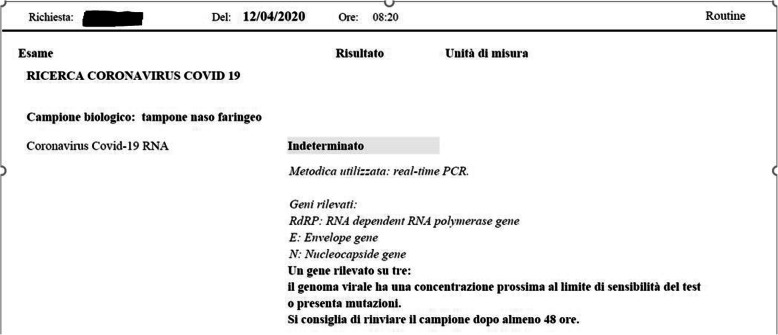
One of the three RT-PCR swabs shows a doubtful result (April 12)

During the hospitalization, three RT-PCR swabs were performed, two had a negative result, and the third a doubtful result (Fig. 4).

**Fig. 5:**
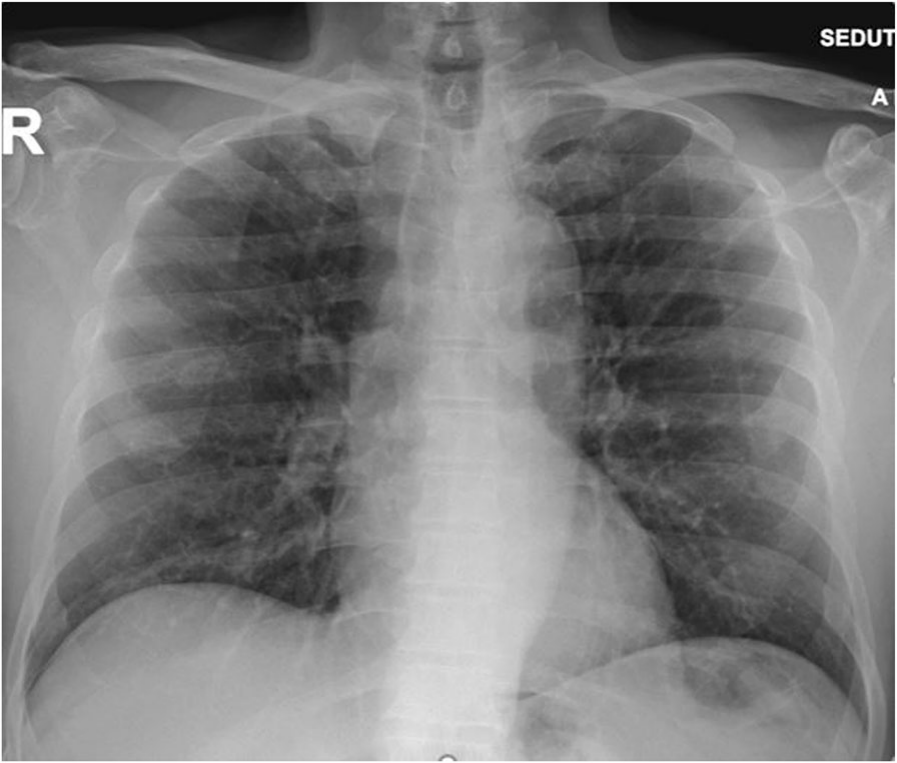
Chest RX, PA projection (April 21). No evidence of pleuro-parenchymal alterations in both lung areas

Despite this, having excluded the bacterial nature of the serological alterations (negative serology for Mycoplasma and Chlamydia), the patient was definitively treated for COVID-19 with an experimental protocol (azithromycin, hydroxychloroquine, heparin) with the resolution of the lung involvement (Fig. 5).

## Discussion

Potentially relevant incidental findings are very common in WB-RM [15]. In this case, in which the patient was completely asymptomatic, in an area with a high prevalence for COVID-19, all sanitation measures were immediately put in place and he was sent to a dedicated CT room. Chest CT is an important method for detecting (symptomatic patients with negative chest X-Ray) and follow-ups in SARS-CoV-2 pneumonia.

Chest CT findings of COVID-19 have been widely reported in the literature [16–20]. On March 13, our patient was probably in a prodromal phase of COVID-19: the patient was only asthenic (symptoms attributable, moreover, to treatment for MM) [21]. After a few days (March 16), he manifested the first symptoms that persisted for another 10 days (March 26 at least). By the time he underwent the WB-MR (March 10) the patient was probably instead in a phase of resolution of the disease, therefore again asymptomatic. The ground glass and crazy-paving CT features could agree with an absorption phase.

Pan et al. [22], in fact, in their study reported a temporal correlation of the chest CT findings, distinguishing the radiological findings in 4 stages:

- Early stage (0-4 days after onset of the initial symptom): there are GGOs, which are the main radiological manifestation distributed subpleural in the lower lobes unilaterally/bilaterally.
- Progressive stage (5-8 days after the onset of the initial symptom): there are widespread GGOs, crazy flooring patterns, and consolidations. The infection rapidly aggravates and extends to a bilateral multi-lobed distribution.
- Peak stage (9-13 days after the onset of the initial symptom): there are still widespread GGOs, crazy flooring patterns, consolidation, and residual parenchymal bands.
- Absorption stage (≥ 14 days after the onset of the initial symptom): a large GGO could be seen as a demonstration of the absorption of consolidation. This absorption phase extends for over 26 days from the start of the initial symptoms. The character and extent of abnormalities beyond 4 weeks remain unknown but one can expect similarities to other acute lung injuries with resolution or residual scar.

This timeline reconstruction would also explain the dubious result of the RNA-PCR test. In particular, it is believed that the RT-PCR test has high specificity but sensitivity has been reported to be between 60 and 70% [23].

Therefore, excluding a diagnosis of COVID-19 requires multiple negative tests, with test kits hardly available or unavailable. In response to lung reports anomalies of CT without positive RT-PCR, the Chinese authorities initially expanded the official definition of infection to include patients with typical CT findings, even with a first negative RT-PCR result [24, 25]. The anamnestic information, the laboratory information, and the radiological features confirm that our patient, even without a positive RT-PCR test, was affected by COVID-19. The absence of symptoms both in the prodromal phase and in the “absorption/resolution” phase is underlined. In the late phase of the disease, the radiological findings, suggestive for COVID-19, were dissociated from the symptoms.

This highlights the key role of the radiologist in detecting occasional findings especially in areas where the coronavirus is present, as its spread can be hidden in patients in whom the disease has not been diagnosed and/or in asymptomatic cluster.

## Conclusion

In consideration to the spreading of a virus, which currently still remains unknown; the right approach is to leave nothing to chance and to pay utmost attention to the management of radiological facilities when treating patients. During this pandemic, any patient should be treated as COVID-19 positive as the possibility to come into contact with asymptomatic patients still remains high.

## Data Availability

The datasets used and/or analyzed in the report are available from the corresponding author on reasonable request.

## Abbreviations

ASCT: Autologous stem cell transplantation
COVID-19: Coronavirus disease 19
GGO: Ground-glass opacity
MM: Multiple myeloma
MR: Magnetic resonance
RT-PCR: Real-time polymerase chain reaction
WB-MR: Whole-body MR
WHO: World Health Organization
